# Implantable cardiac monitor and leadless pacemaker in the management of syncope due to intermittent high-degree atrioventricular block: a case report

**DOI:** 10.1186/s13019-024-02962-x

**Published:** 2024-07-13

**Authors:** Nian Tang, Xiaoxiao Chen, Denghong Zhang, Hongfei Li

**Affiliations:** Geriatric Diseases Institute of Chengdu/Cancer Prevention and Treatment Institute of Chengdu, Department of Cardiology, Chengdu Fifth People’s Hospital (The Second Clinical Medical College, Affiliated Fifth People’s Hospital of Chengdu University of Traditional Chinese Medicine), Chengdu, 611137 China

**Keywords:** Implantable ECG event monitor, Leadless atrioventricular synchronous pacemaker micra AV, High atrioventricular block

## Abstract

**Background:**

Lead dislodgements, tricuspid valve failure, and wound infections are prominent issues addressed by leadless pacemakers (LPM). These devises have emerged as viable alternatives to conventional transvenous pacemakers. LPMs offer minimized complications and effective pacing, particularly beneficial for elderly patients with a low body mass index (BMI) who are at heightened infection of risk. The Micra AV leadless pacemaker was released in the US in 2020, featuring a VDD pacing mode akin to conventional pacemakers. It senses atrial activity to pace ventricular beats while maintaining the natural atrioventricular activation sequence. Micra AV achieves atrioventricular synchronization through mechanical sensing principles. Ongoing research aims to assess its efficacy, implantation feasibility, and clinical safety.

**Case presentation:**

An 83-year-old man with a history of syncope was the focus of this case study. An implantable cardiac monitor (ICM) recorded occasional high-degree atrioventricular block in the patient. Subsequently, the Micra AV was implanted via the left femoral vein, and its settings were adjusted in accordance with data obtained from the ICM. No significant issues regarding pacing threshold or impedance were found during the follow-up examinations post-surgery. Importantly, the patient experienced a noticeable reduction in symptoms compared to before the implantation.

**Discussion:**

This case underscores the significance of ICM monitoring in elucidating cardiac events leading to syncope and guiding appropriate treatment. It also highlights the successful outcomes and reliable implantation of the Micra AV for managing high-degree atrioventricular block. This study contributes to the growing body of evidence supporting the adoption of leadless pacemakers as a viable option for patients requiring cardiac pacing, particularly those vulnerable to complications associated with traditional pacemakers. It provides real-world evidence of Micra AV’s efficacy and safety, further validating its role in clinical practice.

## Background

Leadless pacemakers (LPM) have emerged as an alternative to traditional transvenous pacemakers, effectively avoiding problems such as wound infection, lead dislodgements, and tricuspid valve dysfunction. This is particularly beneficial for elderly patients with a low body mass index (BMI), who are at heightened risk of infection. In 2020, Micra AV, a leadless pacemaker featuring a VDD pacing function (ventricular pacing following atrial sensing, preserving the normal atrioventricular activation sequence), was launched in the United States. Micra AV achieves atrioventricular synchronization using mechanical sensing principles. Currently, its clinical safety, implantation feasibility, and effectiveness are undergoing post-marketing research and data collection.

**Fig. 1:**
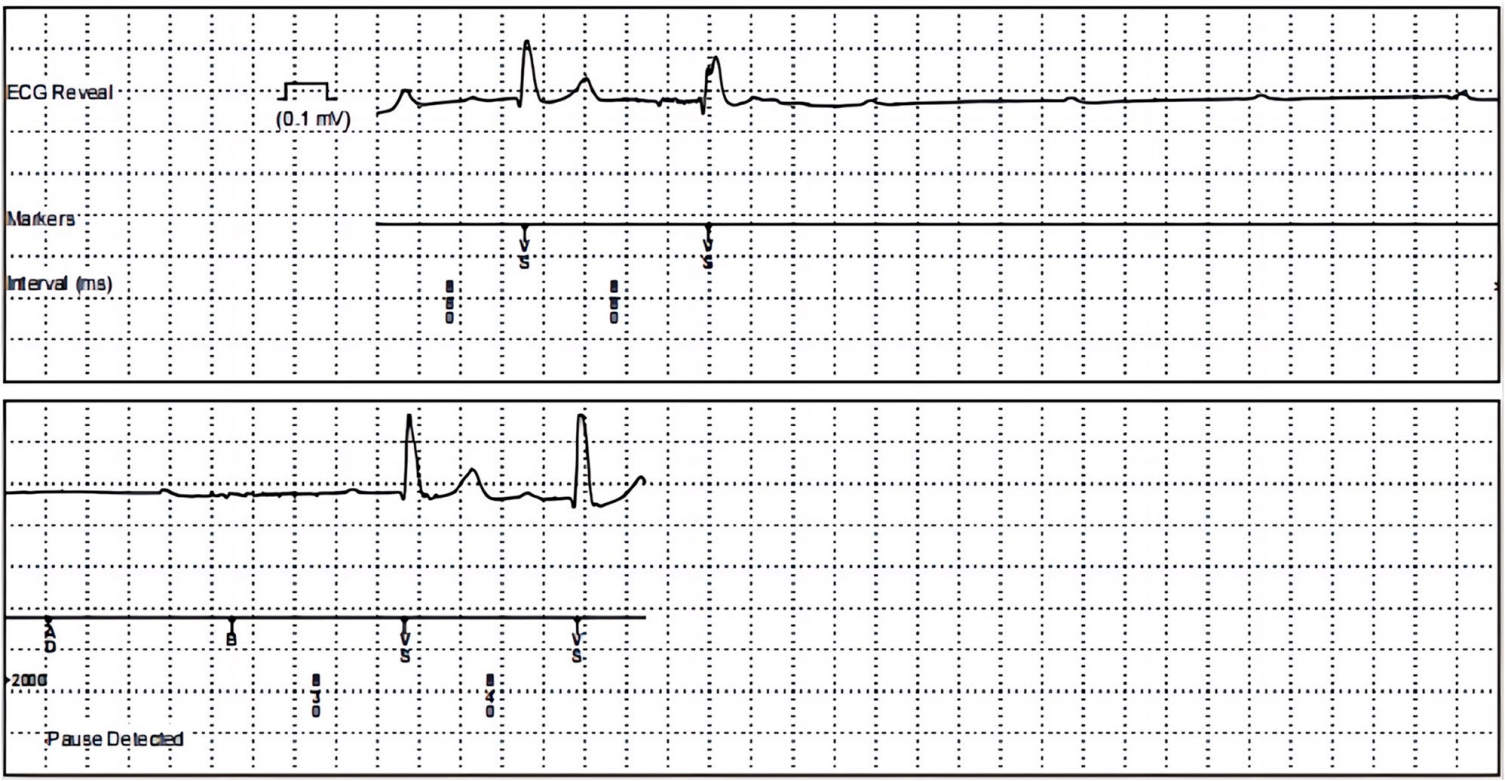
ICM monitors 6:1 height atrioventricular block electrocardiogram

Here, we present the case of an 83-year-old man with a history of syncope, who was found to have intermittent high-degree atrioventricular block using an Insertable Cardiac Monitor (ICM) (Fig. 1). Subsequently, Micra AV was implanted through the left femoral vein, and its parameters were adjusted based on the data collected by the ICM.

### Case introduction

All procedures performed in this study adhere to the ethical standards of the institution and/or National Research Council guidelines, and the principles outlined in the Declaration of Helsinki (2013 revision). Written informed consent was obtained from the patient to publish this case report along with accompanying images. Copies of the written consent are available for review by the editorial board.

The patient, an 83-year-old man, was hospitalized due to recurrent syncope episodes over the past two years. Initially, he experienced sudden dizziness and weakness with subsequent loss of consciousness, followed by spontaneous recovery after a few minutes without significant discomfort. Similar intermittent episodes occurred intermittently thereafter. Echocardiography revealed left atrial enlargement with normal left ventricular systolic function. Coronary angiography indicated coronary atherosclerosis. Head MRI + MRA showed an old lacunar infarct in the left lateral ventricle and pontine area, alongside intracranial atherosclerosis. Electroencephalogram results were unremarkable. Holter ECG monitoring confirmed sinus rhythm with intermittent second-degree type I atrioventricular block, prompting a Class IIa recommendation for pacemaker implantation [1]. Further electrophysiological examination was advised to pinpoint the site of block or to implant an ICM to monitor cardiac events during syncope [2]. The patient opted for ICM implantation, which revealed evidence linking intermittent high-degree atrioventricular block to his syncope episodes. Given the risks of syncope and potential progression to permanent atrioventricular block, the patient’s pacing indication was upgraded to Class I, necessitating pacemaker implantation. Due to the patient’s advanced age, slender physique, limited life expectancy, and low anticipated ventricular pacing requirement, Micra AV was chosen and implanted via femoral vein.

### Surgery procedure

**Fig. 2:**
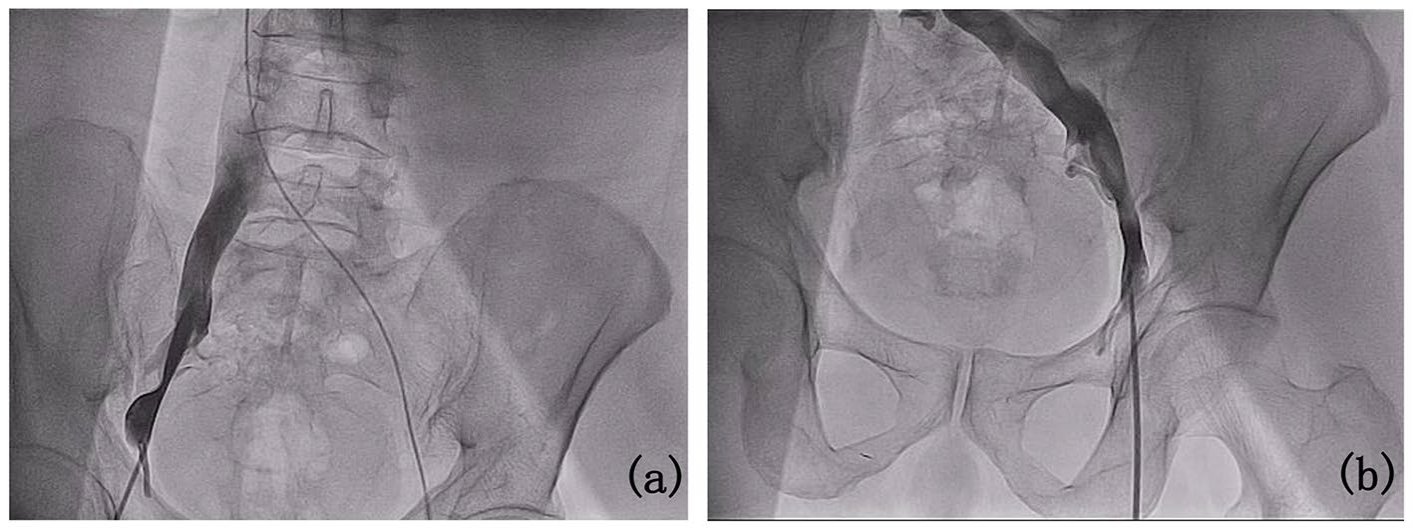
Venography Results. (**a**) Right femoral venography showed mild stenosis of the iliac vein; (**b**) Left femoral venography showed clear blood vessels

**Fig. 3:**
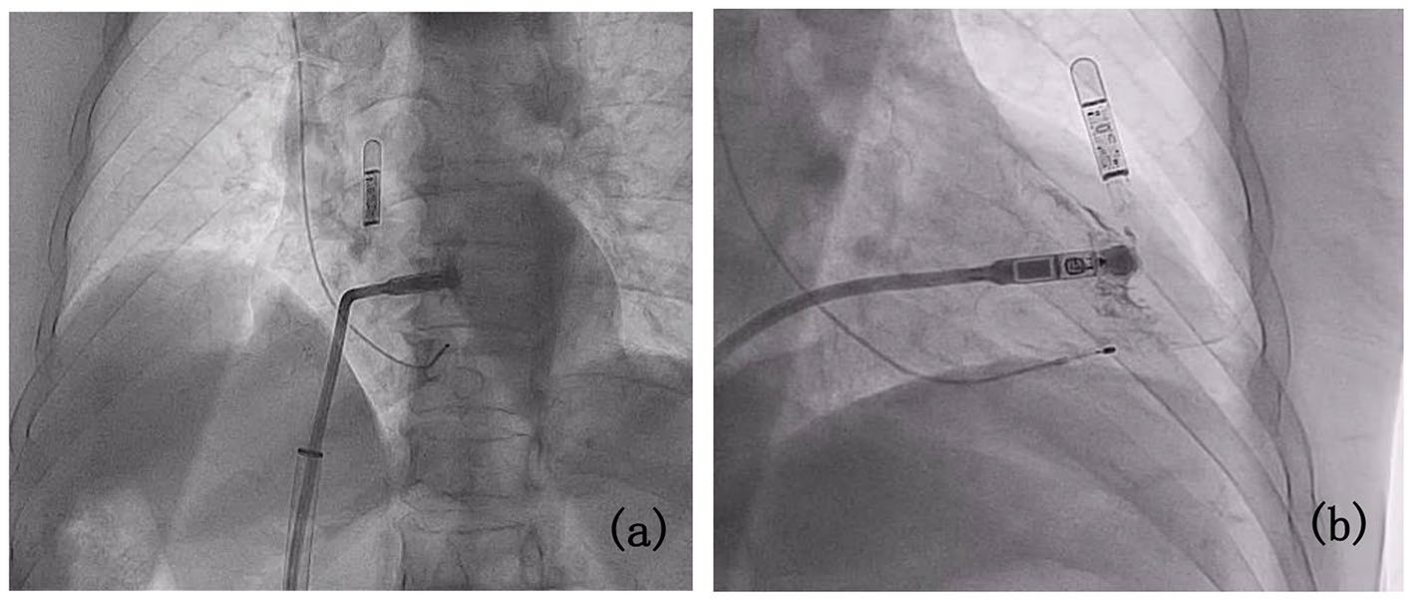
Post-implantation radiographs. Radiographs in the left and right anterior oblique projections (**a** and **b**) following implantation of the leadless pacemaker

Firstly, the left femoral vein was accessed for temporary pacemaker insertion. Subsequently, attempts to dilate the 8 F and 10 F sheaths placed via the right femoral vein revealed mild stenosis of the right lateral iliac vein during femoral venography (Fig. 2a). Due to unsuccessful dilation, the approach was shifted to the left femoral vein. The temporary pacemaker from the left femoral vein was removed, and a new temporary pacemaker was inserted via the internal jugular vein. Left femoral venography confirmed patency of the left femoral vein, external iliac vein, and common iliac vein (Fig. 2b). During the procedure, the patient experienced obvious distension pain and mild vasovagal reaction. Administration of atropine stabilized the patient’s vital signs, enabling successful delivery of the leadless pacemaker system through the sheath, crossing the tricuspid valve into the right ventricle. Contrast agent injection through the delivery system assessed positioning against the right ventricular septum in various body positions (Fig. 3a and b).After satisfactory angiography, Micra AV was released, and a traction test confirmed secure fixation. Three surgeons confirmed stable positioning of the pacemaker head metal winglets in the myocardium. Pacing parameters post-implantation were as follows: threshold 0.63 V / 0.24ms, impedance 680Ω, R-wave sensing 10.7 mV, indicating satisfactory electrical function. The procedure was completed in 60 min without complications. There was no evidence of bleeding or fluid leakage at the puncture site on the first day after surgery. Program-controlled electrical parameters remained stable after the operation. The patient was discharged from the hospital on the second day post-surgery.

**Fig. 4:**
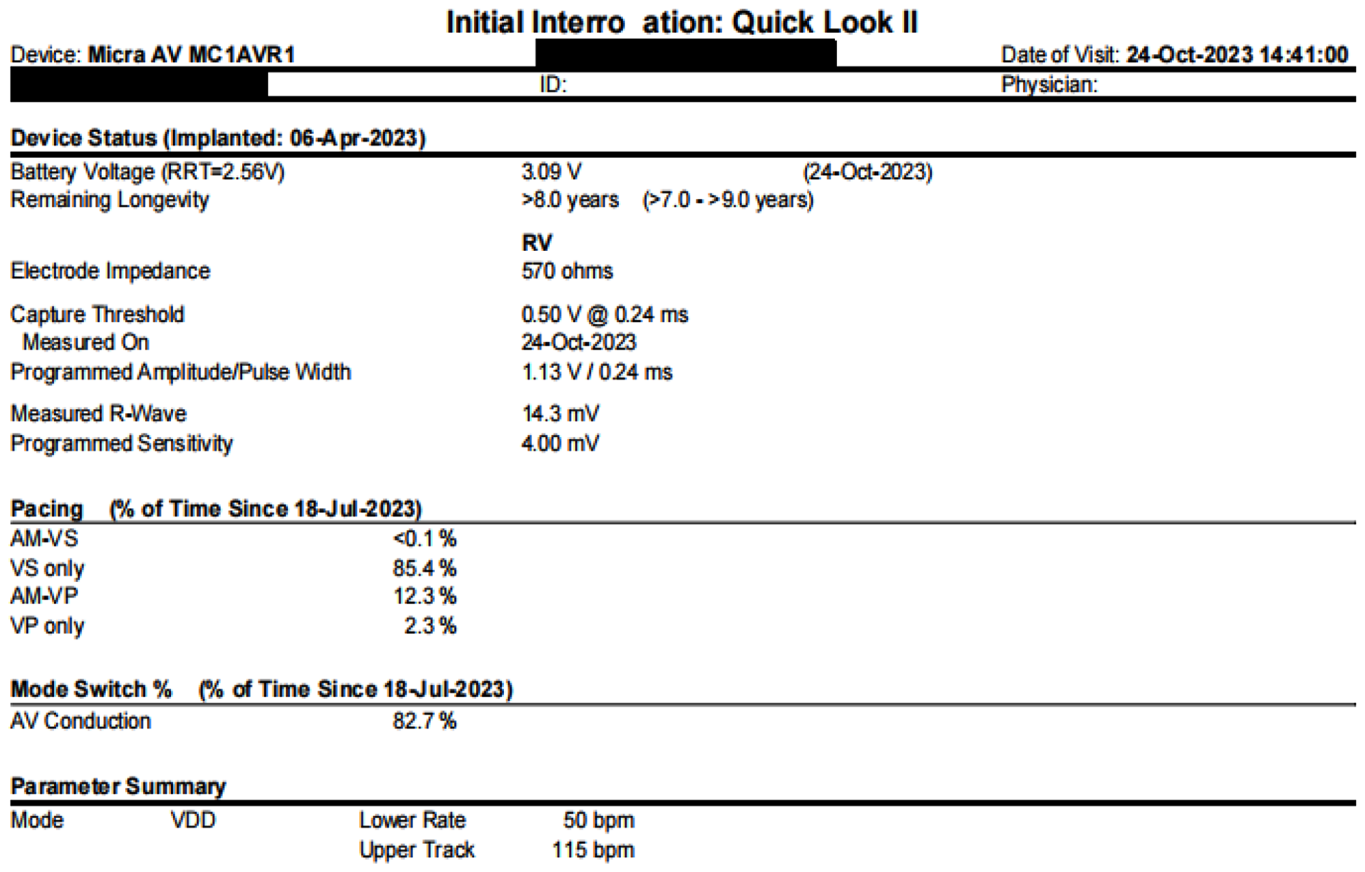
Pacemaker remote interrogation at 3-month follow-up

At the 3-month follow-up, program-controlled examination of pacemaker showed stable pacing thresholds and impedance for Micra AV, with appropriate atrioventricular tracking. Atrial-ventricular (AM-VP) and ventricular pacing (VP) frequencies were 12.3% and 2.3%, respectively (Fig. 4). The patient continues to exhibit intermittent high-degree atrioventricular block as the underlying heart rhythm.

## Discussion

While transvenous pacemakers remain a well-established treatment for chronic arrhythmias, complications related to eads and/or pocket management significantly restrict their utility. Leadless pacemakers have emerged as an alternative to traditional transvenous pacemakers. Our case identified the underlying cause of syncope through ECG events recorded by an ICM implantation, highlighting the feasibility and safety of dual-chamber leadless pacemaker implantation in elderly patients.

Unlike traditional pacemakers, safety and effectiveness of leadless pacemaker have been improved [3]. Micra AV is particularly suitable for patients at high risk of infection such as the elderly, those with severe wasting, abnormal venous anatomy, diabetes mellitus, and those undergoing hemodialysis. Micra AV utilizes a built-in triaxial acceleration sensor to detect blood flow acceleration during atrial contraction, thereby triggering ventricular pacing and providing atrioventricular synchronous pacing. The clinical study MARVEL II enrolled a total of 75 patients [4], with 40 cases included in the effectiveness analysis. The results showed that among patients with normal sinus rhythm and complete atrioventricular block, the median resting atrioventricular synchronization rate was 94.3%, with an average atrioventricular synchronization rate of 89.2%. These findings validate the efficacy of Micra AV in managing patients with atrioventricular block. With the emergence of Micra AV, more patients with atrioventricular block can now benefit from leadless pacemakers [5]. The decision to use the Micra AV leadless pacemaker in this case was guided by a comprehensive evaluation of the patient’s risk factors, anatomical considerations, and the potential clinical advantages of a leadless system. We believe that this approach yielded the most facorable outcome for the patient, aligning with our goals to minimize complications and maximize therapeutic efficacy. In summary, the patient, aged 83, diagnosed with intermittent high-degree atrioventricular block and normal sinus rhythm, alongside a slender physique, limited life expectancy, and low anticipated ventricular pacing requirements. The use of a VDD type leadless pacemaker like Micra AV was deemed particularly beneficial in this context.

Julius Jelisejevas et al. ‘s study [6] involved 143 patients who underwent continuous Micra TPS implantation, with 87% implanted via the right femoral vein and 13% via the left femoral vein. Left femoral vein access for Micra TPS implantation is safe, effective, comparable to traditional right femoral vein access, achieving excellent implantation success rates. In our case, left femoral vein access was chosen because the right iliac vein presented limitations, precluding passage. The implantation of Micra AV proceeded smoothly, with successful delivery, release, and secure fixation in the lower right ventricular space. The patient experienced transient mild vasovagal reaction due to distending pain during the procedure, but recovered promptly. Typically, conventional Micra TPS implantation through the right femoral vein targets median septum of the right ventricle. However, in this instance using the left femoral vein approach, the final positioning was in the middle to lower septum, influenced by the placement of the 27F delivery sheath closer to the right atrial lateral wall. The angle through the tricuspid ring differed, resulting in a distinct final device position.

In addition, the cardiac event recorded by ICM in this case was intermittent high-degree atrioventricular block. Consequently, the conduction mode switch was activated during parameter adjustment, transitioning the Micra AV from VDD pacing mode to VVI + mode, the pacemaker provides ventricular pacing support at a rate of 40 beats per minute when atrioventricular conduction recovers. The conduction function was monitored every 8 h, and if a ventricular rate greater than 40 beats per minute was detected, the pacemaker maintained VVI + mode. This configuration conserves battery power and extends the pacemaker’s lifespan while ensuring pacing needs are met, specifically tailored for patients with intermittent atrioventricular block. The parameters of the Micra AV were set based on ECG events recorded by the ICM, enhancing the safety and efficacy of pacing support provided to the patient.

We present the case of an elderly patient with atrioventricular block who underwent successful implantation of Micra AV via a left femoral vein approach following detection of an ECG event by an ICM. This case underscores the diagnostic utility of implantable ECG event monitors in investigating unexplained syncope episodes. It also validates the safety and effectiveness of the new Micra AV generation in elderly patients, expanding indications to include those with sinus rhythm and AVB.

## Data Availability

The datasets used and/or analysed during the current study are available from the corresponding author on reasonable request.

## Abbreviations

LPM: Leadless Pacemakers
ICM: Insertable Cardiac Monitor
